# DNA vaccines expressing conserved elements provide potent and broad immune responses

**DOI:** 10.1186/1742-4690-9-S2-O67

**Published:** 2012-09-13

**Authors:** GN Pavlakis, V Kulkarni, A Valentin, M Rosati, NY Sardesai, B Mothe, C Brander, S LeGall, DB Weiner, M Rolland, JI Mullins, BK Felber

**Affiliations:** 1 Inovio Pharmaceuticals, Inc., Blue Bell, PA, USA; 2 IrsiCaixa-HIVACAT, Barcelona, Spain; 3 Ragon Institute of MGH, MIT and Harvard, Boston, MA, USA; 4 University of Pennsylvania, Philadelphia, MD, USA; 5 U.S. Military HIV Research Program, Rockville, MD, USA; 6 University of Washington, Seattle, WA, USA; 7 Frederick National Laboratory for Cancer Research, Frederick, MD, USA

## Background

Immunodominance and sequence diversity are major hurdles in the development of effective HIV vaccines. We tested the hypothesis that a vaccine candidate composed of strictly Conserved Elements (CE) of the HIV proteome excluding the variable regions would help overcome problems of viral sequence diversity and potential negative effects of immunodominance. Seven CE were identified in p24^gag^. Vaccination of macaques with p55^gag^DNA failed to elicit cellular or humoral immune responses to the CE, while epitopes outside of the CE were immunogenic.

## Methods

Two HIV p24^gag^CE DNA plasmids were generated, providing potential epitopes found in >99% of all HIV-1 M group sequences. DNA vectors, optimized for gene expression were used to immunize mice and macaques by IM injection followed by in vivo electroporation.

## Results

Vaccination with p24^gag^CEvac DNAs elicited potent, cross-clade cellular and humoral immune responses. Highly cytotoxic CE-specific T cells, capable of Granzyme B production and degranulation, were generated. Importantly, boosting of the CEvac-primed macaques with p55^gag^DNA greatly augmented the CE-specific cellular responses (up to 10-fold) as well as humoral responses, despite the failure of p55^gag^DNA vaccine to induce de novo CE-specific responses. CEvac DNA prime-p55^gag^DNA boost in mice led to similar conclusions. Interestingly, mapping analysis showed differential increase of the CE-specific responses by the p55^gag^DNA boost, demonstrating changed hierarchy of CE responses in macaques.

## Conclusion

Vaccination with the p24^gag^CEvac DNA overcame the problem of diversity by generating strong cross-clade Gag-specific immune responses, and of immunodominance, eliciting responses to subdominant but highly conserved elements, and also by broadening the p55^gag^DNA induced immunity. p55^gag^DNA did not induce de novo responses to the CE, but was able to significantly boost pre-existing CE-induced responses and alter the hierarchy of these responses. Translation of this concept into clinical trials may elicit cross-clade cellular immune responses against components of the viral proteome with limited capacity for immunological escape.

